# Evaluation de la qualité de la prise en charge de la dyspnée par les médecins généralistes à Parakou en 2013

**DOI:** 10.11604/pamj.2015.22.350.7649

**Published:** 2015-12-11

**Authors:** Léopold Houétondji Codjo, Serge Hugues Dohou, Anthèlme Agbodandé, Bastu Mohamed Karimou, Armand Finangnon Wanvoegbe, Angelo Cossi Attinsounon, Adébayo Cossi Alassani, Martin Dèdonougbo Houénassi

**Affiliations:** 1 Unité d'Enseignement et de Recherche en Cardiologie, Faculté de Médecine, Université de Parakou, Parakou, Benin; 2 Hôpital d'Instruction des Armées de Parakou, Parakou, Bénin; 3 Service de Médecine Interne, Centre National Hospitalier Universitaire Hubert Koutoukou Maga, Cotonou, Bénin; 4 Service de Médecine du Centre Hospitalier Universitaire Départemental du Borgou, Parakou, Benin; 5 Unité d'Enseignement et de Recherche en Cardiologie, Faculté des Sciences de la Santé, Université d'Abomey-Calavi, Bénin

**Keywords:** Dyspnée, prise en charge, Afrique, Dyspnea, management, Africa

## Abstract

**Introduction:**

La dyspnée est un symptôme qui peut être l'expression de pathologies potentiellement grave et urgentes. Notre objectif était d’évaluer la qualité de la prise en charge de la dyspnée en milieu hospitalier à Parakou.

**Méthodes:**

Il s'agissait d’étude rétrospective portant sur tous les patients admis dans les centres hospitaliers de Parakou pour dyspnée non traumatique entre le 1er Février 2012 et le 31 Mai 2013. Les recommandations tunisiennes sur la prise en charge de la dyspnée aux urgences ont été utilisées comme référentiel d’évaluation. Chaque étape de la démarche médicale a été notée et la prise en charge était bonne lorsque la note obtenue au score était entre 51 et 100. Les autres variables utilisées étaient la qualification du soignant principal, les antécédents du patient et les données cliniques et paracliniques.

**Résultats:**

Sur les 11101 patients reçus aux urgences on a dénombré 328 cas de dyspnée (2,9%). La PEC était assuré principalement par les cardiologues (55,9%) et les médecins généralistes (29,2%). La qualité de la prise en charge par les généralistes était bonne chez 73,2%. Les facteurs associés à la bonne qualité de PEC étaient: la recherche des antécédents médicaux (84,4% vs 15,6%; p < 10-4), la description des caractéristiques cliniques de la dyspnée (94,1% vs 5,9%; p < 10-4) et la réalisation de l'examen physique complet (86,8% vs 13,2%; p < 10-4).

**Conclusion:**

A Parakou en 2013 près d'un généraliste sur trois gère mal la dyspnée. Cette mauvaise gestion est liée à une mauvaise démarche médicale.

## Introduction

La dyspnée est de fréquence variable en pratique médicale courante [1]. Elle a des étiologies multiples potentiellement graves [2–4]; ce qui rend sa prise en charge délicate même dans les centres bien équipés. De nombreux algorithmes décisionnels sont proposés pour optimiser la prise en charge de la dyspnée. Cependant, on note une incertitude dans le diagnostic étiologique de la dyspnée aux urgences dans environ 50% des cas en France [5]. Dans le contexte des pays en voie de développement, ces algorithmes décisionnels montrent leur limite d'usage en raison de l'inaccessibilité à de multiples examens permettant une recherche étiologique précise: dosage des D-dimères, le dosage des BNP, dosage de la troponine, étude des gaz de sang, réalisation de l'angioscanner thoracique. A Parakou, la pratique médicale a été confrontée à une forte fréquence de la dyspnée gérée dans un contexte de plateaux techniques insuffisants. Après deux ans d'exercice cardiologique dans ces conditions, il nous a paru utile d’évaluer les résultats de la prise en charge de la dyspnée et plus spécifiquement par les médecins généralistes.

## Méthodes

Cette étude s'est déroulée dans les services d'accueil des urgences, de médecine interne, de cardiologie à l'Hôpital d'Instruction des Armées de Parakou (HIA-Parakou) et dans les services d'Anesthésie-Réanimation et Urgences (SARU), de médecine et spécialités médicales au Centre Hospitalier Départemental du Borgou (CHD-B). Il s'est agi d'une étude documentaire qui a analysé de manière rétrospective et systématique tous les dossiers médicaux de patients admis pour dyspnée non traumatique dans ces deux hôpitaux. La période d’étude était de seize (16) mois allant du 1^er^ Février 2012 au 31 Mai 2013. La collecte des données a été faite à l'aide de fiches de dépouillement, après accès au dossier des patients. Tous les patients reçus pour dyspnée non traumatique étaient inclus dans notre étude. Etaient exclus, ceux dont les dossiers n'ont pas été retrouvés ou incomplets et ceux âgés de moins de quinze (15) ans. La variable dépendante était la qualité de la prise en charge de la dyspnée. Pour réaliser cette évaluation de qualité, nous nous étions référés aux recommandations proposées par le ministère de la santé publique tunisien sur la prise en charge de la dyspnée aux urgences [6]. Les différentes parties de la démarche diagnostique ont été cotées de 0 à 100 points. Nous avons accordé les points les plus élevés à l'interrogatoire (25 points) compte tenu de son importance dans l'orientation étiologique et dans la prise en charge de la dyspnée [2]. La cotation et son interprétation ont été présentées dans leTableau 1. Les données ont été vérifiées, codifiées et analysées à l'aide du logiciel Epi Info7. La moyenne, la médiane et l’écart type ont servi à décrire les variables quantitatives. Nous avons évalué le risque d'erreur à un seuil de 5%. Une valeur de p <0,05 était considérée comme statistiquement significative. Un accord commun a été obtenu des autorités administratives et des chefs des différents services du CHD-B et de l'HIA-Parakou. Les fiches d'enquête ont été remplies dans l'anonymat à l'aide d'un code secret d'identification garantissant la confidentialité des données.

**Tableau 1:** Score d’évaluation de la qualité de la prise en charge de la dyspnée par les médecins généralistes dans les hôpitaux de la ville de Parakou du 1er Février 2012 au 31 Mai 2013

Items	Points
**Données de l'identité:**	
Âge précisé	3
Sexe précisé	3
Adresse précisée	2
Professsion précisée	2
**Antécédents médicaux:**	
Antécédents cardiovasculaires et autres ntécédents recherchés	5
Facteurs de risque cardiovasculaire recherchés	5
**Modalité d'admission précisée:**	5
**Données de l'anamnèse (Caractéristiques cliniques de la dyspnée):**	
Date et mode de début recherchés	5
Mode d’évolution recherché	5
Types de dyspnée recherchés	10
Signes associés recherchés	5
**Les données de l'examen physique:**	
Apprèciation de l’état général	3
Prise des constantes	3
Examen physique des différents appareils éffectué	4
**Hypothèse diagnostique:**	
Une hypothèse au moins émise	5
Mise en condition réalisée si hypothèse de pathologie grave	5
**Examens complémentaires demandés:**	
Biologie	4
Radiologie/Echographie cardiaque	3
Electrocardiogramme	3
**Un diagnostic est retenu:**	10
**Un traitement est institué:**	10
**TOTAL**	**100**

**Appréciation de la description de la dyspnée:** 0 ≤ score ≤ 16 pas bonne; 17 ≤ score ≤ 25 bonne

Appréciation de la qualité de la prise en charge: 0 ≤ score ≤ 50 pas bonne ; 51 ≤ score ≤ 100 bonne

## Résultats

### Fréquence de la dyspnée

**Figure 1:**
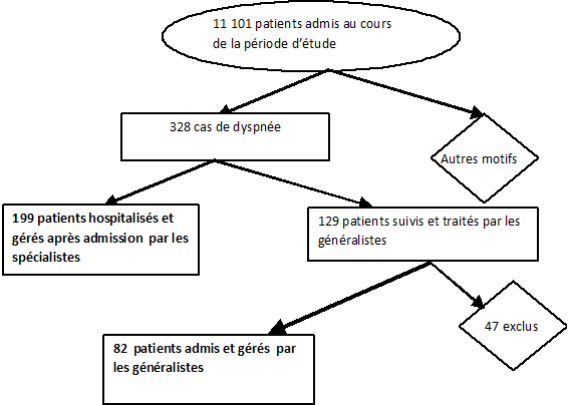
Diagramme de flux présentant la sélection des patients pour l’évaluation de la prise en charge hospitalière de la dyspnée par les médecins généralistes à Parakou du 1er Février 2012 au 31 Mai 2013

Sur la période d’étude, 11101 patients ont été admis dans les services des urgences des deux hôpitaux (5481 au CHD-B et 5620 à l'HIA-Parakou). Parmi eux, nous avons répertorié 328 cas de dyspnée (98 patients au CHD et 230 patients à l'HIA), soit une prévalence de 2,9% des admissions aux services d'urgence. Cent quatre-vingt-dix-neuf(199) ont été hospitalisés et gérés par les médecins spécialistes. Le Tableau 2 présente la répartition des patients selon la nature du médecin soignant. Les médecins généralistes ont géré aux urgences 129 patients dont 47 exclus pour dossiers incomplets ou non retrouvés. Le diagramme de flux de la Figure 1 présente la sélection des patients.

**Tableau 2:** Répartition des patients victimes de dyspnée à Parakou du 1^er^ Février 2012 au 31 Mai 2013 selon la nature du soignant principal

Qualification du soignant	Effectif	Pourcentage
Médecins généralistes	129	39,3
Médecins spécialistes		
Cardiologues	157	47,9
Internistes	24	7,3
Réanimateurs	17	5,2
Neurologue	1	0,3
Total	328	100,0

### Caractéristiques générales de la population

La moyenne d’âge était de 49,9 ± 18,9 ans avec des extrêmes de 16 ans et de 91 ans. Dans notre série, 39 patients étaient de sexe masculin (47,3%) et 43 de sexe féminin (52,7%) soit une sex-ratio égale à 0,9. En ce qui concerne la profession, les ménagères, les fonctionnaires et les commerçants constituaient les classes les plus représentées dans les proportions respectives de 22,1%; 18,5% et 14,2%. Le mode d'admission était une référence dans 23,5% des cas.

Evaluation de la prise en charge de la dyspnée par les généralistes

### Données de l'interrogatoire

La nature de la profession avait été précisée chez 57 patients sur 82 (69,5%); la recherche des antécédents médicaux a été complète chez 56 patients (68,3%). Les facteurs de risque de maladie respiratoire avaient été recherchés chez 56 patients (68,3%). En ce qui concerne la description de la dyspnée, le mode d'installation a été précisé chez tous les patients. Le type de la dyspnée a été précisé chez 75 patients (91,5%); la date de début chez 65 (79,3%); le mode d’évolution chez 30 (36,6%) et les signes associés chez tous les patients. Ainsi la qualité de la description clinique de la dyspnée, a été bonne chez 59 patients (71,9%), et pas bonne chez 23 patients (20,7%).

### Données de l'examen clinique

Les données de l'examen clinique étaient incomplètes chez 35 patients soit 42,7% des cas pris en charge par les généralistes. L'appréciation de l’état général n'avait pas été précisée chez ces 35 patients; les constantes végétatives n'avaient pas été prises chez neuf patients (11%); l'examen des différents appareils (cardio-vasculaire, pleuro-pulmonaire et neurologique) n'avait pas été réalisé chez 5 patients (6,1%).

### Examens complémentaires

Lorsque l'indication était posée, les examens paracliniques étaient effectivement demandés dans 64,6% pour la biologie (hémogramme, ionogramme sanguin, créatininémie, glycémie) 40,2% pour la radiographie thoracique, 36,6% pour l’électrocardiogramme et 7,3% pour l’échographie Doppler cardiaque. Les taux de réalisation étaient respectivement de 88,7%; 84,8% 86,7% et 50%. Au moins un diagnostic étiologique de dyspnée été évoqué chez tous les patients. Tous les traitements prescrits par les médecins étaient basés sur un diagnostic retenu dans tous les cas. Conformément à la grille d’évaluation, la qualité de la prise en charge a été “pas bonne” dans une proportion de 26,8% et “Bonne” chez 73,2% des médecins généralistes.

### Facteurs associés à la qualité de la prise en charge

La bonne qualité de la prise en charge est significativement associée à la profession, la recherche des antécédents médicaux des patients, la bonne description de la dyspnée, et à la réalisation d'un examen physique complet (Tableau 3).

**Tableau 3:** Facteurs associés à la qualité de la prise en charge de la dyspnée par les médecins généralistes à Parakou du 1^er^ Février 2012 au 31 Mai 2013

	Qualité de la prise en charge (n = 281)		p
	Pas bonne N(%)	Bonne N(%)	
**Profession recherchée**			
Oui	16(66,7)	219(85,2)	0,016
Non	8(33,3)	38(14,8)	
**Recherche des antécédents**			
Complète	12(50,0)	217(84,4)	<10^−4^
Incomplète	12(50,0)	40(15,6)	
**Description de la dyspnée**			
Bonne	11(45,8)	242(94,1)	<10^−4^
Mauvaise	13(54,2)z	15 (5,9)	
**Examen physique**			
Complet	6(25,0)	223(86,8)	<10^−4^
Incomplet	18(75,0)	34(13,2)	
**Diagnostic final évoqué**			
Oui	24(100,0)	252(98,1)	0,31
Non	0	5(1,9)	

## Discussion

Pour évaluer la qualité de la prise en charge de la dyspnée en milieu hospitalier à Parakou, nous avons réalisé une enquête rétrospective basée sur le dépouillement des dossiers médicaux des patients. Le caractère rétrospectif de cette recherche nous a permis d’évaluer l'attitude réelle des généralistes face à la dyspnée. L'utilisation d'un référentiel africain [6] a rendu notre jugement réaliste et objectif. Dans notre étude la prévalence de la dyspnée était de 2,9% des admissions au service d'accueil des urgences. Cette prévalence corrobore celle de 3% retrouvée par Jean-Yves B. et al en suisse [1] et se rapproche de celle retrouvée par Ray P. et al [2] en France (5%). Elle est nettement inférieure à celle retrouvée par Gombet T. et al [3] à Brazzaville (36,6%) dont les travaux ont porté essentiellement sur les patients présentant une urgence cardiovasculaire. L’âge moyen de 49,9 ± 188,9 ans dans notre série se rapproche de celui rapporté par Malas O. et al [8] en Turquie (54,4 ±16,1 ans). En revanche, dans un pays développé comme la France, l’âge moyen était plus élevé: 80 ± 9 ans [2]. La différence observée serait liée au fait qu'en Afrique, non seulement l'espérance de vie est moins élevée mais aussi que ce symptôme relève plus de la plainte de sujets jeunes [16]. Les médecins généralistes n'avaient pas recherché la profession des patients dans 30,5% des cas. D'après l'Institut scientifique de la santé publique Belge [9], le diagnostic de l'asthme professionnel par exemple est fréquemment raté. L’établissement d'un diagnostic correct, condition à une prise en charge adéquate de la dyspnée demande donc l'analyse de l'histoire professionnelle des patients. Les facteurs de risque cardiovasculaire n’étaient pas recherchés dans 31,7% des cas. Selon Ennezat PV. et al [10], l'existence d'un antécédent de cardiopathie ou de pneumopathie chronique fait évoquer en première intention sa décompensation. Elle permet également d'estimer la gravité potentielle d'une dyspnée. Pour Bertrand E. et al [11], le pronostic d'une dyspnée est conditionné par le terrain et les comorbidités. On en déduit que la recherche des antécédents médicaux a non seulement un intérêt diagnostique mais aussi thérapeutique et pronostique. Les caractéristiques cliniques les mieux renseignés étaient le type de dyspnée, les signes associés et le mode évolutif. La date de début n’était pas renseignée chez un patient sur cinq. De nombreux auteurs parmi lesquels Berney JY. et al [12] en Suisse, Somrani N. et al [6] en Tunisie, rapportent que le type de la dyspnée, le mode de début et le mode d’évolution sont des éléments d’évaluation de la gravité immédiate et des situations de menaces vitales. La recherche optimale des caractéristiques de la dyspnée est une condition impérative à la démarche diagnostique. Ces résultats montrent que les médecins généralistes doivent être plus rigoureux et exhaustifs dans la description des caractéristiques cliniques de la dyspnée. Selon Morelot-Panzini C. [13], l'interrogatoire est la clé de voûte de la démarche diagnostique devant une dyspnée. Pour Caumon L. [9] il est un temps essentiel de la prise en charge de la dyspnée. Armand-Perroux A. et al [14] rapporte que l'analyse anamnestique reste la pierre angulaire de l’étude de la gravité d'une embolie pulmonaire.

Les constantes végétatives n'avaient été prises que dans 1 cas sur 10; l'examen physique des différents appareils n'avait pas été réalisé dans 6,1% des cas. Dans les référentiels de prise en charge de la dyspnée aux urgences élaborés par le ministère tunisien de la santé en 2010 [6], l'examen physique est obligatoire pour orienter la démarche diagnostique et la demande d'examens complémentaires spécifiques. Pour Caumon L. [8], l'examen physique cardio-pulmonaire fournit un arbre diagnostique dans la prise en charge. Il doit donc être complet chez tout patient reçu aux urgences pour dyspnée. La biologie avait été majoritairement demandée par les médecins généralistes devant la radiographie thoracique, l’électrocardiogramme et l’échographie Doppler cardiaque. Pourtant, la radiographie thoracique, l’électrocardiogramme et l’échographie Doppler cardiaque sont des bilans de première intention dans la prise en charge de la dyspnée [6, 13]. Ce faible taux de demande pourrait être l'expression d'une méconnaissance des recommandations sur la prise en charge de la dyspnée par les généralistes. Le taux de réalisation de ces bilans dans un ordre croissant était de 50% pour l’échographie Doppler cardiaque; de 84,8% pour la radiographie thoracique; de 86,7% pour L'ECG et de 88,7% pour la biologie. L'insuffisance de la réalisation des bilans pourrait s'expliquer par la faible couverture sociale (absence de sécurité sociale et de mutuelles) des patients qui sont pour la plupart d'un niveau socio-économique modeste [15]. Les difficultés d'approvisionnement en réactifs et les problèmes de maintenance des appareils utilisés dans les laboratoires seraient également des facteurs réduisant le taux de réalisation des examens complémentaires. En ce qui concerne la prise en charge, un patient sur quatre n'avait pas eu une bonne prise en charge par les médecins généralistes. Les insuffisances retrouvées pour les connaissances et attitudes pratiques de ces médecins dans la démarche diagnostique et le non respect des recommandations pourraient justifier ces résultats. Les facteurs associés à la bonne qualité de la prise en charge que nous avons retrouvés étaient: la recherche de la profession du patient dans la rubrique identité, la recherche des antécédents médicaux, la recherche des caractéristiques cliniques de la dyspnée, la réalisation d'un examen physique complet. Le respect de la démarche médicale est capital pour une meilleure prise en charge. En effet Morelot-Panzini C. [13] dans une étude sur le diagnostic et les principes de prise en charge d'une dyspnée a conclu qu'une démarche diagnostique systématique et une évaluation multidimensionnelle permettent d'appréhender plus précisément les mécanismes et la sévérité de ce symptôme respiratoire et ainsi de guider la prise en charge. Selon Armand-Perroux A. et al [14], une démarche inappropriée expose le patient à une mauvaise prise en charge.

## Conclusion

En milieu hospitalier à Parakou, la prise en charge de la dyspnée était assurée par les cardiologues suivis des médecins généralistes. La qualité de la prise en charge par les généralistes n’était pas bonne chez tous les patients. Cet état de cause serait favorisé par une méconnaissance des recommandations de prise en charge qu'il urge de corriger par l'organisation des séances de recyclage et de mise à jour des médecins généralistes
